# Editorial: Genetic regulation of mitosis and ploidy in cancer

**DOI:** 10.3389/fgene.2023.1264772

**Published:** 2023-08-30

**Authors:** Somsubhra Nath, Pallavi Agarwal

**Affiliations:** ^1^ Institute of Health Sciences, Presidency University, Kolkata, West Bengal, India; ^2^ Amity Institute of Molecular Medicine and Stem Cell Research, Amity University Uttar Pradesh, Noida, India

**Keywords:** cancer, chromosomal instability (CIN), genetic instability, aneuploidy, mitosis, prognosis (carcinoma)

The phrase “*Genetic regulation of mitosis and ploidy in cancer*” comes with a disclaimer that cancers, of different origins, frequently show deregulation of genomic stability. The proper execution of mitosis successfully generates two euploid progeny cells, while deregulated mitosis in malignant cells makes aneuploidy a hallmark of cancer. Mitosis provides a stage where cyclins, cyclin-dependent kinases, motor proteins, and other proteins, participating in the process, interplay in a time-dependent manner towards maintaining a unidirectional flow of cell division. Other cellular events namely, DNA replication, DNA damage repair, centrosome duplication, and spindle polymerization also contribute towards this goal. Genetic as well as epigenetic modification of the key mitotic players are hypothesized, examined, and subsequently validated through various studies. Indeed, newer findings are emerging with intricate details, articulating better-targeted approaches against mitotic gene candidates, as potential therapeutic measures for various malignancies. This Research Topic has been put together to highlight new and upcoming molecular aberrations in mitotic and non-mitotic genes and epigenetic changes that regulate mitotic progression and ploidy of malignant cells, playing a crucial role in carcinogenesis.

The regulation of mitosis largely depends on the spindle assembly checkpoint (SAC). At its molecular level, the mitotic checkpoint complex (MCC) blocks the activity of anaphase-promoting complex/cyclosome (APC/C) until all the dividing cells attain the amphitelic spindle-kinetochore attachments. While functioning, APC/C acts in assistance with one of its co-activators, namely, Cdc20 or Cdh1. Greil et al., in their review under this section, connected the data, published so far, on the different aspects of Cdc20 and Cdh1. This review ended up with the translational relevance of these two molecules, along with APC/C, towards the clinical management of cancer. Moreover, this review highlighted the findings on SAC in promoting aneuploidy and other chromosomal instabilities (CIN). One such example of CIN is genomic copy number variation (CNV). In a brief research report on a study cohort of chronic myeloid leukemia (CML), Zhang et al. found Philadelphia chromosome is the only detectable CIN in a maximum number of cases. A few cases were detected bearing CNVs, which were further associated with the adversities of the disease. Similarly, through extensive research conducted on study cases of primary diffuse B-cell lymphoma of the central nervous system, several candidate CNVs and mutations were identified by Zhu et al. These mutational landscapes play a considerable role in the pathogenesis and clinical determination of this category of malignancy. Impairment of mitotic checkpoints, DNA damage checkpoints, and DNA repair machinery leads to genomic instability, the major driving force towards malignancy. The high mutational and epigenetic reprogrammed landscape of malignant cells also promotes immune evasion of tumor cells. Lv et al. explored the prognostic and predictive value of long non-coding RNAs (lncRNAs) related to genetic instability by analyzing lncRNA expression in breast cancer patients with high genetic instability (high mutation group) versus low genetic instability (low mutation group). Comparing lncRNA expression with overall patient survival, the authors constructed a genetic instability-related 7 lncRNA model including U62317.4, SEMA3B-AS1, MAPT-AS1, AC115837.2, LINC01269, AL645608.7, and GACAT2. This model successfully predicted the survival outcomes of patients in two independent cohorts. The authors proposed that high-risk lncRNAs might have promoting effects on breast cancer. Differentially expressed genes associated with high-risk groups are enriched in immune pathways including the B-cell receptor signaling pathway. By generating protein-protein interaction networks, hub genes of the adaptive immune pathway and B-cell receptor signaling pathway were found to be correlated with the overall survival of breast cancer patients. The authors confirmed high expression of CXCL8, a chemokine known to induce immune cell infiltration, in high-risk lncRNA patient groups. Interestingly, CXCL8 expression positively correlated with enhanced M2 macrophage infiltration mediating local immunosuppression. Thus, the authors suggested how high mutational rates and genetic instability may affect immune cell infiltration and finally influence the tumor immune microenvironment. Thus, early detection and intervention in genetic instability may represent an effective measure for improving the prognosis of breast cancer patients. Another study presented in this Research Topic by Tao et al. focused on microtubule-associated molecular motor kinesin family protein and explored its prognostic value in glioma patients. Kinesin Family Member 18A (KIF18A) participates in the cell cycle and in mitotic metaphase and anaphase, however, its function in brain cancer is largely unknown. This study highlighted KIF18A as a new promising prognostic biomarker that correlates with uncontrolled mitosis and immune infiltration in malignant gliomas. The authors presented a correlation of high expression of this kinesin molecule with increased age (>60 years), high grade (4th grade), wildtype status of 1p/19q co-deletion, and poor survivability of glioma patients. The authors compared the differentially expressed genes (DEGs) in high versus low KIF18A-expressing glioma patients and the DEGs were enriched in cell cycle, microtubule organizing, and cell division pathways. High KIF18A-expressing patients had an altered tumor microenvironment with positive infiltration of TH2 cells, macrophages, neutrophils, eosinophils, and alpha-dendritic cells. The data presented in the manuscript revealed KIF18A as an independent risk factor for poor prognosis of glioma patients. Using clinical samples and cell lines, the authors further validated the correlation of high KIF18A expression with advanced tumor grades of glioma patients. Thus, the study suggested KIF18A as a new molecular target that regulates mitosis and tumor immune infiltration and might serve as a potential prognostic biomarker for glioma malignancies.

ABC transporters are established cancer drivers playing a major role in drug resistance and the development of stemness in tumor cells. A mechanistic understanding of ABC transporters in mitotic proliferation and tumor cell migration in pancreatic cancer remains unknown. The study from Zheng et al. published in this Research Topic identified ABC transporter ABCA12 as a key regulator of the AKT signaling pathway which in turn modulates proliferation and migration of pancreatic cancer cells. By analyzing 30 clinical samples of pancreatic cancers and adjacent matched controls, the authors provided IHC-based expression data of ABCA12, which confirmed significantly high ABCA12 expression in cancer tissues compared to adjacent controls. Further, the authors identified a close correlation between high expression of ABCA12 with tumor grade and lymph nodes. The data on ABCA12 silenced pancreatic cancer cells depicted lower metastatic and invasion abilities, enhanced proliferation defect, and induction of apoptosis. Further, ABCA12 knockdown mitigated PI3K/AKT pathway by inactivating AKT phosphorylation, thus regulating the proliferation and migratory potential of tumor cells. This study presented an example of the carcinogenic mechanism of ABC transporters regulating cancer hallmarks, other than chemotherapeutic and multidrug resistance. Thus, the study provided a new target for pancreatic therapy and emphasizes exploiting the role of ABCA12 in other cancers as a potential cancer-promoting driver gene.

The articles in this Research Topic emphasize that early detection and intervention in genetic instability might effectively improve cancer prognosis, which would be worth testing in various cancers. Also, the finding of high-risk lncRNAs coinciding with high mutational load and their role in promoting tumor immune infiltration is a new concept. This issue also reports the non-canonical roles of motor proteins in tumor infiltration and ABC transporter proteins in cellular proliferation, which would be worth exploring. We hope that the readers would find this Research Topic on mitosis and aneuploidy in cancer informative and exciting to read.

